# The evolving landscape of health and social care integration: in search of a unifying theory

**DOI:** 10.3389/fmed.2025.1464699

**Published:** 2025-07-17

**Authors:** Kheng Hock Lee, Chien Earn Lee

**Affiliations:** ^1^Duke-NUS Medical School, Singapore, Singapore; ^2^Singhealth Community Hospitals, Singapore, Singapore; ^3^Singapore General Hospital, Singapore, Singapore; ^4^Singapore Health Services Pte Ltd, Singapore, Singapore

**Keywords:** social prescribing, complexity, complexity science, health system, care model

## Abstract

This paper explores the co-evolution of theoretical paradigms and care models within health and social care through the lens of complexity science. It argues that this co-evolution, characterized by a dynamic interplay between abstract principles and tangible practices, propels health systems toward greater human-centeredness and interconnectedness. Theories, acting as “attractors,” shape the landscape of possible care models, while the implementation and evaluation of these models, in turn, refine theoretical understanding. This continuous feedback loop, driven by emergent properties within the complex system of healthcare, fosters a dynamic evolution toward more holistic and effective care. The paper proposes a unifying framework to understand this ongoing process, emphasizing the interconnectedness of individual, community, and societal well-being. While further research is needed to validate this conceptual framework, it offers a valuable lens for analyzing historical trends and guiding future developments in health and social care.

## Introduction

Complex health needs, marked by the co-occurrence of physical, mental, and social challenges, have become increasingly prevalent, underscoring the limitations of traditional, compartmentalized healthcare systems that are ill-equipped to address these multifaceted issues (1). Traditional healthcare systems, designed to address issues in isolation, are struggling to cope with the rising tide of complex needs. An aging population, which inherently contributes to a greater prevalence of co-morbidities, often results in individuals navigating a web of intertwined health and social care needs that defy compartmentalized approaches. In response to this pressing need, the healthcare landscape has witnessed the evolution of integrated care models that seek to bridge the divide between health and social domains, offering a more holistic and person-centered approach to addressing the complex needs of individuals and communities (2, 3).

Health and social care systems increasingly mirror complex adaptive systems, comprised of diverse agents—patients, providers, policymakers, and community actors—each operating with varying goals and levels of influence. These systems are characterized by nonlinearity, interdependence, and emergent behavior, making them resistant to traditional top-down control and prediction. Complexity science provides a valuable lens through which to interpret this evolving landscape, highlighting the importance of relationships, feedback loops, and adaptability in producing meaningful and sustainable change (4).

While momentum builds around new care models designed to integrate care, the field still needs a unifying theoretical framework to guide its continued advancement. This commentary proposes that the ongoing evolution of care models, reflecting a series of paradigm shifts in healthcare as described by Thomas Kuhn, has been unfolding since the postwar period (5). Each era tends to champion a dominant model, treating it as *the* solution, much like a passing fad. However, reflecting on history reveals that these models often have roots in past approaches and are destined to evolve into future paradigms. This constant evolution is driven by the changing landscape of population health needs and the growing understanding of health as a multidimensional construct.

## The WHO era and the shift toward holism

The World Health Organization’s 1948 definition of health as “a state of complete physical, mental and social well-being and not merely the absence of disease or infirmity” marked a pivotal turning point in the conceptualization of health (6). This holistic definition emerged in the shadow of global conflict and unprecedented medical atrocities, standing as a direct rebuke to the prevailing biomedical model.

In the decades preceding the establishment of the WHO, there was a growing emphasis on the mechanistic reductionism of disease, often depersonalizing the individual patient and transforming them into an object in the pursuit of scientific advancement, all in the name of objectivity. This trend, while yielding important medical breakthroughs, also created an enlarging ethical blind spot, culminating in the horrific human experimentation perpetrated by doctors and researchers. The world, reeling from the horrors of these acts, recognized the urgent need for a more humane and holistic vision of health—one that placed equal emphasis on healing the well-being of the whole person, not just rectifying defects of the physical body. The WHO definition, with its emphasis on “complete” well-being and its inclusion of mental and social dimensions, offered a powerful counter balance to the dangers of a purely depersonalized view of medicine.

## The rise of the biopsychosocial model and social determinants

Building on the WHO’s holistic foundation, the biopsychosocial model, proposed by George Engel in the 1970s, further expanded the conceptual framework for understanding health. Engel challenged the prevailing biomedical paradigm, arguing that the biological aspects of disease could not be fully understood without accounting for the influential roles of psychological and social factors (7). The model recognized that the experience and outcome of illness is shaped not only by physiological processes, but also by the individual’s cognitive and emotional responses, as well as the broader social contexts and determinants that shape their overall health and well-being. This more comprehensive view of health and illness paved the way for the development of integrated care models that seek to address the complex interplay of biological, psychological, and social issues that affect wellbeing.

While the biopsychosocial model represented a significant shift in thinking, its translation into practice often remained limited to a technical, systems-based approach focused on “quality improvement” metrics. This approach, while aiming to enhance efficiency and effectiveness, often overlooked the importance of genuine human connection and personalized care. It remained a mechanistic model, akin to a process improvement strategy in manufacturing, prioritizing measurable outcomes over the nuanced experiences of individuals navigating complex health and social challenges. The emergence of the social determinants of health (SDOH) framework further exposed the limitations of purely process improvement approaches. SDOH illuminated the profound influence of factors beyond the individual, such as:

1. *Socioeconomic Conditions:* Poverty, income inequality, and lack of access to education and employment opportunities significantly shape health outcomes.
2. *Environmental Factors:* Exposure to pollution, unsafe neighborhoods, and limited access to healthy food choices directly impact well-being.
3. *Social and Community Networks:* Social isolation, discrimination, and lack of social support contribute to a range of physical and mental health issues.
4. *Cultural Beliefs and Practices:* Cultural norms and beliefs influence health behaviors, access to care, and perceptions of illness.

This framework underscored a critical point: true health equity cannot be achieved by simply improving healthcare systems. It demands addressing the root causes of health inequities embedded in the fabric of society (8).

While care models like the chronic care model and patient-centered medical home have emerged and often incorporated principles of social determinants of health (SDOH), they have often struggled to fully embrace a person-centered approach. The challenge remains: how do we move beyond simply acknowledging SDOH to developing care models that genuinely empower individuals and communities to thrive within their unique social and environmental contexts?

## Re-personalizing care

A parallel and influential movement toward a more intentional person-centered approach was taking shape within the field of psychotherapy. Carl Rogers, in the mid-20th century, pioneered person-centered therapy, a radical departure from the prevailing psychoanalytic paradigms. Rogers’ work emphasized the importance of empathy, unconditional positive regard, and genuineness in the therapeutic relationship, empowering individuals to take an active role in their own healing and growth (9).

In the mid-1980s, Edward Deci and Richard Ryan were developing self-determination theory (SDT), a theory of motivation that emphasizes the importance of autonomy, competence, and relatedness for well-being and optimal functioning. SDT posits that individuals are naturally motivated to grow and thrive when these basic psychological needs are met (10).

The integration of person-centered principles, stemming from humanistic psychology and Self-Determination Theory, significantly transformed the landscape of healthcare delivery. No longer were patients viewed as passive recipients of standardized treatments. Instead, care models began to prioritize collaboration, shared decision-making, and a deep respect for individual values and preferences. This philosophical shift challenged the traditional paternalistic approach, urging healthcare providers to view their patients as active participants in their own healing journeys. The focus expanded beyond simply treating diseases to encompass a more holistic understanding of well-being, acknowledging the interconnectedness of mind, body, and environment.

While structural and institutional integration are important there is a need to consider a personal and relational based approach to health-social integration. This means understanding “disease management” from the individual’s perspective: how they feel about their condition, its impact on their lives, and their chosen approach to coping. Simultaneously, health promotion efforts should address the individual and community values that influence health behaviors. Recognizing that values are shaped by various life factors, such as family, social circles, education, media, and personal experiences, can lead to more effective health promotion strategies (11).

This philosophical shift toward person-centered care manifested in tangible changes to care models. The emphasis moved from standardized protocols to personalized care plans, recognizing the uniqueness of each individual’s experience of illness and their path to recovery. Therapeutic relationships, characterized by trust, empathy, and genuine human connection, became paramount, replacing the often transactional nature of traditional clinical encounters. This human-centric approach led to the emergence of innovative care models like the Patient-Centered Medical Home (12). While challenges remain in fully realizing person-centered care within complex healthcare systems, this philosophical shift has undoubtedly laid the groundwork for a more humane, compassionate, and effective approach to healthcare delivery.

## Re-centering on the community

Complementing this individual focus, the emergence of asset-based community development (ABCD) in the early 1990s provided a comprehensive framework for community health that moves beyond a traditional deficit-based perspective. ABCD emphasizes identifying, mobilizing, and leveraging the existing strengths, assets, and resources within communities to create positive change and improve health outcomes (13). By recognizing and actively engaging these community-based assets, ABCD empowers residents to play an active role in addressing local health challenges. This approach fosters resilience, self-determination, and collective efficacy, enabling communities to harness their own capacities and social capital to address the underlying determinants of health. The ABCD model represents a paradigm shift in community health, moving away from an exclusive reliance on external interventions and toward a more collaborative, community-driven approach that builds upon the inherent strengths and resources within neighborhoods and communities.

Complementing the individual focus of person-centered care, asset-based community development emerged as a powerful framework for fostering community health. Shifting away from a deficit-based perspective that solely focused on problems, ABCD emphasized recognizing and leveraging the inherent strengths, assets, and resources already present within communities. This approach empowered residents to become active agents of change, fostering resilience, self-determination, and a sense of collective efficacy. By mobilizing existing assets, such as local organizations, community leaders, and the skills and talents of residents, ABCD facilitated community-driven solutions to address the social, economic, and environmental factors influencing health (14).

This shift toward asset-based approaches had a profound impact on the practice of medicine. Healthcare providers began to recognize the limitations of solely focusing on individual patients within the confines of clinical settings. They increasingly acknowledged the powerful influence of community environments on health outcomes and the importance of partnering with community-based organizations to address the social determinants of health. This collaboration extended the reach of healthcare beyond the traditional medical model, connecting patients with resources and support systems within their communities to address needs related to housing, food security, social connection, and more. This integrative approach, combining individual-level care with community-level support, reflects a growing understanding of the interconnectedness of individual and community well-being.

Community-based approaches like ABCD exemplify the principle of emergence, a cornerstone of complexity science. Rather than relying solely on top-down interventions, these models recognize that sustainable change often arises from bottom-up innovation, where local actors mobilize existing assets and relationships. This aligns with complexity’s view that solutions should be context-sensitive, adaptive, and responsive to the specific configurations of needs, strengths, and histories present within each community.

## The co-evolution of theories and practice

Care models, in essence, represent the practical embodiment of evolving theoretical perspectives, arising from a dynamic and often overlapping process of applying these theories to real-world challenges. Just as a theory of change outlines a desired outcome and then maps out the necessary steps and interventions to achieve it, the evolution of care models mirrors this process. As our understanding of health, well-being, and human behavior deepens through theoretical advancements, so too do our approaches to care delivery evolve. This constant interplay between theory and practice manifests as new care models that appear to become dominant at different time periods.

The Chronic Care Model, gaining traction in the late 1990s, emphasized a proactive, patient-centered approach to managing chronic conditions. It sought to transform the delivery of healthcare for those with long-term illnesses by promoting coordinated, evidence-based care that engaged patients as active partners in managing their own health. The model highlighted the importance of a prepared, proactive healthcare team that worked collaboratively with informed, activated patients to improve outcomes and quality of life for those living with chronic diseases (15).

Concurrently, Local Area Coordination emerged in the 1990s, focusing on building partnerships between health and social services to address the broader determinants of health (16). These localized, place-based approaches aimed to coordinate and integrate various services and community resources to better support individuals and families with complex needs. Integrated care models, seeking to better coordinate care across different healthcare settings and providers, also gained prominence in the 2000s as a way to improve patient outcomes and experiences by breaking down silos between primary, secondary, and community-based care.

The Patient-Centered Medical Home (PCMH) model, which emerged as a key approach in primary care, emphasizes a comprehensive, team-based approach to care delivery. At its core, the PCMH model focuses on fostering strong, collaborative relationships between patients and their healthcare providers. By promoting a partnership between the patient and a dedicated care team, the PCMH model aims to enhance care coordination, improve access to services, and empower patients to actively participate in managing their own health and well-being (17). The team-based approach brings together various healthcare professionals, including physicians, nurses, social workers, and care coordinators, to provide coordinated, comprehensive, and personalized care tailored to the individual’s unique needs and preferences. This holistic, patient-centered model represents a shift away from the traditional, fragmented healthcare system toward a more integrated and collaborative model of primary care delivery.

More recently, Social Prescribing has gained recognition as an innovative approach to addressing the complex social, emotional, and practical needs of individuals. This model goes beyond the traditional biomedical focus of healthcare by connecting people to a wide range of community-based resources and activities tailored to their unique circumstances and preferences. Through Social Prescribing, individuals struggling with issues in the social determinants such as social isolation, loneliness, or other non-clinical challenges can be referred by their healthcare providers to local services and support groups that can help improve their overall well-being (18). By facilitating access to community-based interventions such as exercise classes, arts and crafts workshops, volunteering opportunities, or peer support groups, Social Prescribing empowers individuals to actively engage with their local communities and build meaningful connections. By expanding the scope of care beyond clinical settings, Social Prescribing represents yet another paradigm shift toward a care model that integrates the multiple theories such as the social determinants of health, asset based community development and the biopsychosocial model of health.

These models, while apparently distinct, share a common thread of recognizing the interconnectedness of individual, social, and environmental factors in shaping health outcomes. They represent a series of paradigm shift in healthcare, moving away from a reductionist, disease-focused approach toward a more comprehensive understanding of health and well-being. By acknowledging the complex interplay between biological, psychological, social, and environmental determinants, these models underscore the need for integrated, person-centered care that addresses the multifaceted nature of an individual’s health challenges. This evolution in theoretical frameworks has paved the way for the development of innovative care models that aim to bridge the gaps between various healthcare and social service sectors, fostering a more holistic and collaborative approach to supporting individual and community health.

**Figure 1:**
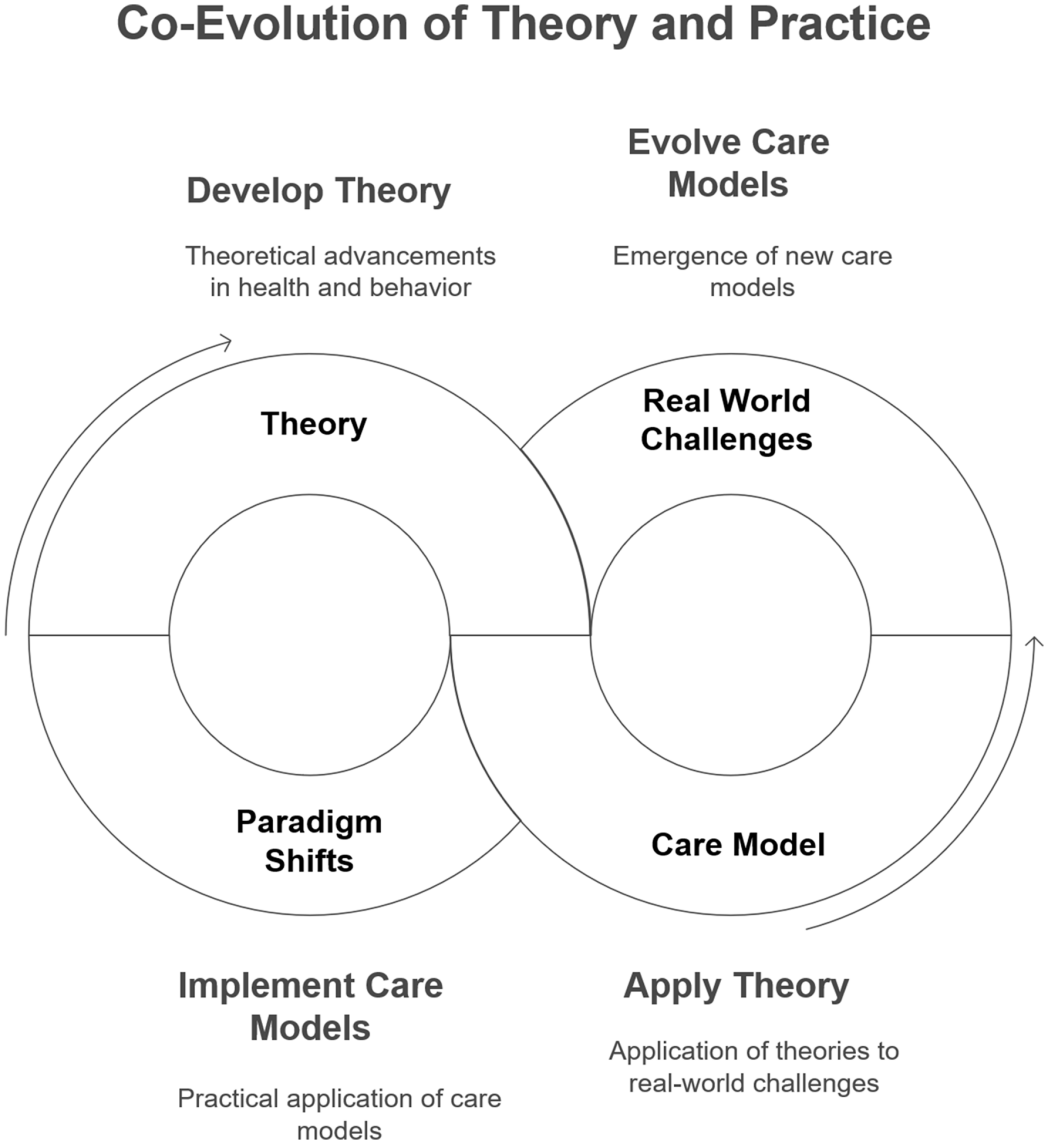
The figure illustrates the dynamic relationship between theory and care models. The application of a theory gives rise to care models, which, when implemented in practice, encounter real-world challenges and anomalies. Over time, these issues lead to a paradigm shift, prompting the development of a new or evolved theory.

This process exemplifies the nonlinear dynamics central to complexity science. Theories and care models do not evolve in a sequential or isolated manner; instead, they influence each other through recursive feedback loops. A new model may arise from a theoretical framework, but its real-world implementation often produces insights that refine or even challenge the originating theory. This co-evolution reflects the system’s capacity to learn and adapt, demonstrating that progress in healthcare is not a linear trajectory but a dynamic and iterative process shaped by ongoing interactions. Created to illustrate the idea of co-evolution of theory and practice proposed in the paper (Figure 1).

## Discussion

Thomas Kuhn’s concept of scientific paradigms (19) provides a compelling lens through which to view the evolution of health and social care. Kuhn argued that scientific progress is not linear but rather occurs through shifts in paradigms—fundamental frameworks of understanding that guide research and practice. Similarly, the theoretical underpinnings of health and social care have undergone paradigm shifts, with each new perspective building upon and sometimes challenging preceding ones. The biopsychosocial model expanded upon the limitations of the purely biomedical model, while the social determinants of health framework further broadened the understanding of health influences. This evolution of thought mirrors Kuhn’s notion of paradigms evolving over time, with each successive paradigm encompassing a more comprehensive and nuanced understanding. The emergence of care models alongside these theoretical advancements aligns with Kuhn’s idea that paradigms shape the tools and approaches used in a field. The increasing emphasis on person-centeredness, community assets, and holistic well-being in these models reflects a broader paradigmatic shift in healthcare toward a more humanistic construct of health.

Just as in biological evolution where earlier adaptations remain relevant for survival, older theories in health and social care retain their value and utility. The biomedical model, while considered incomplete in a holistic view of health, remains essential for driving deep scientific understanding and technological advancements in medicine. New theories, rather than completely replacing older ones, often build upon the foundations laid by their predecessors. Similarly, innovative care models often incorporate and adapt elements from previous models, demonstrating an evolution of thought and practice. To achieve effectiveness in health and social care, a systems thinking approach is crucial, recognizing the interconnectedness of various components and their influence on the overall system. Cultivating a higher order of systems thinking, informed by a unifying theory, allows us to view the present through the lens of both past and future, anticipating challenges and opportunities. This foresight enables greater adaptability in the face of a rapidly changing landscape, ensuring the optimal fitness and resilience of our health and social care systems. It reduces the risk reactionary response that result from cognitive dissonance triggered by anomalies that challenge our preferred paradigm.

Adopting a systems thinking perspective reveals how health and social care systems are composed of interconnected subsystems that influence and reshape one another. Adaptive capacity—the system’s ability to change in response to internal and external pressures—is essential for resilience. Complexity science suggests that promoting diverse forms of feedback, learning, and experimentation can strengthen this capacity, enabling systems to remain responsive in the face of uncertainty and continual change.

In this regard, our definition of health may also need to evolve to take into account the interaction of the individual with his/her environment and the reality that with an aging population and rising prevalence of chronic diseases, “a state of complete physical, mental and social well-being” may not be possible for many. A more meaningful goal may be Huber et al.’s definition of “Health as the ability to adapt and to self-manage in the face of social, physical and emotional challenges” (20).

Health and social care systems are not static entities but rather dynamic, adaptive systems driven by a continuous co-evolution of theoretical paradigms and care models. This co-evolution, fueled by a reciprocal interplay between abstract principles and tangible practices, propels the system toward greater human-centeredness and interconnectedness. Just as with other complex adaptive systems, they are comprised of numerous heterogeneous agents – patients, providers, community organizations, policymakers – each making decisions and taking actions within their local contexts. These decisions, shaped by individual experiences and interactions, contribute to the system’s overall evolution, often in non-linear and unpredictable ways. This inherent complexity reminds us that solutions cannot be imposed from the top down but must emerge organically from within, acknowledging the unique circumstances and history of each community. The fundamental principles of this change appear to follow these principles:

1. *Dynamic Interplay*: Theoretical frameworks and care models are not static but engage in a continuous dance of mutual influence and adaptation.
2. *Paradigm Progression*: Theoretical understanding evolves through a process of expansion and refinement, with new paradigms encompassing broader perspectives on health and well-being, particularly emphasizing social and environmental determinants.
3. *Co-Emergence:* Care models emerge as practical embodiments of evolving theoretical principles, translating abstract concepts into tangible interventions and delivery systems.
4. *Human-Centric Trajectory*: This co-evolutionary process inherently steers health and social care systems toward greater human-centeredness, recognizing the individual, community, and societal interconnectedness of well-being.

This potential unifying theory, while requiring further development and empirical validation, offers a framework for understanding the historical progression and future direction of health and social care. It emphasizes the interconnectedness of theory and practice, highlighting the continuous striving toward a more holistic, equitable, and human-centered approach to care.

## Data Availability

The original contributions presented in the study are included in the article/supplementary material, further inquiries can be directed to the corresponding author.
